# An Integrated Nanocomposite Proximity Sensor: Machine Learning-Based Optimization, Simulation, and Experiment

**DOI:** 10.3390/nano12081269

**Published:** 2022-04-08

**Authors:** Reza Moheimani, Marcial Gonzalez, Hamid Dalir

**Affiliations:** 1 Ray W. Herrick Laboratories, School of Mechanical Engineering, Purdue University, West Lafayette, IN 47907, USA; rezam@purdue.edu (R.M.); marcial-gonzalez@purdue.edu (M.G.); 2 Department of Mechanical and Energy Engineering, Indiana University-Purdue University, Indianapolis, IN 46202, USA

**Keywords:** proximity sensor, artificial neural network, multi-objective optimization, genetic algorithm, capacitance, carbon nano tubes

## Abstract

This paper utilizes multi-objective optimization for efficient fabrication of a novel Carbon Nanotube (CNT) based nanocomposite proximity sensor. A previously developed model is utilized to generate a large data set required for optimization which included dimensions of the film sensor, applied excitation frequency, medium permittivity, and resistivity of sensor dielectric, to maximize sensor sensitivity and minimize the cost of the material used. To decrease the runtime of the original model, an artificial neural network (ANN) is implemented by generating a one-thousand samples data set to create and train a black-box model. This model is used as the fitness function of a genetic algorithm (GA) model for dual-objective optimization. We also represented the 2D Pareto Frontier of optimum solutions and scatters of distribution. A parametric study is also performed to discern the effects of the various device parameters. The results provide a wide range of geometrical data leading to the maximum sensitivity at the minimum cost of conductive nanoparticles. The innovative contribution of this research is the combination of GA and ANN, which results in a fast and accurate optimization scheme.

## 1. Introduction

Thanks to the fascinating and broad applications, wearable electronics grow swiftly and require mainly a very efficient sensing system [1,2,3,4,5,6]. Fabrication and design of these systems require a balance between system functionality and cost/energy reduction [7,8,9]. The recent employment optimization methods in the energy consumption of sensory designs are highlighted, which also has motivated researchers to identify more efficient sensors. However, not many optimization strategies are available in the sensor literature nowadays [10,11,12,13,14,15]. Among commercial sensors used in wearable smart devices, flexible nano-based sensors whose sensing materials are nanoparticles such as carbon nanotubes (CNTs), graphene, and metal nanowires become appealing and valuable because they are skin-friendly and durable to withstand mechanical damage [16,17,18,19,20,21].

Proximity sensors are especially demanding candidates for nondestructive measurement of collision prevention in biomedical industries [22,23,24,25,26,27,28,29]. Consequently, it is essential to detect the presence of an object without making contact. Although some optical, ultrasonic, and inductive-based models for proximity sensing have been developed, capacitive-based sensors have a simpler design, easier readout, and a wider range of functionality for metallic and non-metallic targets [30,31,32]. The design of a capacitive proximity sensor should be informed by fundamental electrostatic theories and the tradeoff of sensitivity, resolution, and energy consumption. Although theoretical analyses are somewhat simplified, with some idealized assumptions and strict boundary conditions, and all sensor responses cannot be completely stated, such models still contribute to optimizing sensor parameters to obtain the highest design priority for a practical application. Similarly, studies on wireless sensor networks have proposed an optimization formulation based on experiments and achieved a tradeoff between sensing coverage and energy consumption [33,34]. Recently, optimization of motion sensors was carried out by developing an integrated electromechanical model to predict and simulate the operation of a prototype [10]. For the first time, they directly addressed all device parameters, regardless of their importance. However, a comparative study of capacitive sensors, which benefits of machine learning as a powerful tool, is of great need in wearable electronics.

This paper presents a novel procedure for finding nanocomposite proximity sensors’ optimal size and properties. The nanocomposite sensor has a nanostructure simpler than that of previous studies [18,21,35,36,37,38,39], but in our previous studies [40], there was an intricate microstructure in which CNTs were utilized as a reinforcement in the mold substrate thermoplastic polyurethane (TPU). Our primary results captured the initial testing validation of a capacitive-based sensor (experiment), and later this sensor was simulated by solving the partial differential Laplace equation [41]. Specifically, it was shown that the behavior becomes very sensitive in the specific range of the active materials (CNTs) used as reinforcements in the TPU matrix. Furthermore, other parameters, including geometry, resistivity, dielectric, energy consumption, and frequency, also impact the sensor’s sensitivity.

Our proposed geometrical sensing system was modeled in MATLAB as a Fourier series that uses the key parameters as inputs [41]. One of the main requirements for optimization process is a low runtime. However, this analytical model results in extremely time-consuming simulations, making the optimization process impossible. An Artificial Neural Network can instead provide a black-box model (or function) that works similar to the original MATLAB model and can solve the problem in milliseconds.

Hence, this work represents a Genetic Algorithm (GA) dual-objective optimization process to simultaneously achieve the maximum sensitivity and the minimum cost of nanocomposite materials. The proposed approach builds a black-box model by training an artificial neural network (ANN) with 1000 samples with arbitrary inputs and outputs. Then, the black-box model is employed as the fitness function of a genetic algorithm for a dual-objective optimization of the sensor sensitivity and cost of active materials (CNTs). Consequently, a two-dimensional Pareto Frontier and the scatter distribution of decision variables are employed to find the optimum value or range of decision variables that lead to maximum sensitivity and minimum cost. The primary objectives and novelties of this study are as follows:

- To propose an integrated proximity sensing system that is capable of detecting objects in a wide range by means of active materials.
- To train an ANN black-box model that substitutes the original analytical model and thus makes the GA optimization feasible.
- To implement a dual-objective optimization of sensitivity and cost and later present a two-dimensional Pareto Frontier of the optimum solutions.
- To compare the best solutions for different CNT percentages and illustrate the effect of CNT on the optimum sensitivity and cost.
- To find the effect of different decision variables on each other and on the objective functions using two-by-two scatter distributions.
- To simulate the optimum film sensor using the closed-form analytical model and validate the GA optimization process.

## 2. Model and Experiment Validation

### 2.1. Background

**Figure 1:**
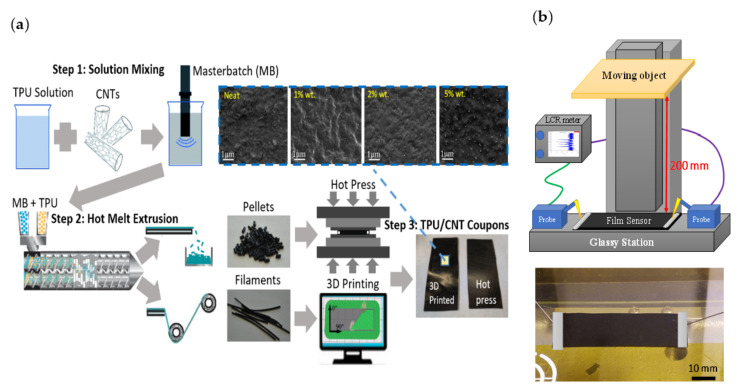
(**a**) Proposed method to process-manufacture TPU with CNT nanocomposites. Step 1: Solution mixing of TPU and CNTs using pro sonication to prepare 10 wt% TPU/CNT masterbatch. Step 2: Melt extrusion to synthesize 1–5 wt% TPU/CNT Pellets and Filaments. Step 3: Hot press of TPU/CNT coupons. SEM images of neat TPU, 1, 2, and 5 wt% TPU/CNT coupons. (**b**) Schematic illustration of a TPU/CNT proximity sensor setup [40].

Figure 1 shows a schematic illustration of the proposed nanocomposite to be used as a nano-based proximity sensor. The device consists of a flexible rectangular-shaped nanocomposite film with two probes (needles) of tungsten mounted across its ends. The narrow strip of two ends was then coated with conductive silver paste. A dielectric (thermoplastic polyurethane) acts as a substrate to restore charges and it is reinforced with Carbon Nano Tubes (CNTs) to make the dielectric conductive. The two electrodes are then connected through an external circuit to an impedance measurement setup. When the film sensor is subjected to the vertical movement of an object, the electric field among the probes is stolen from its surface, and its surfaces charges migrate to the surface of the object.

**Figure 2:**
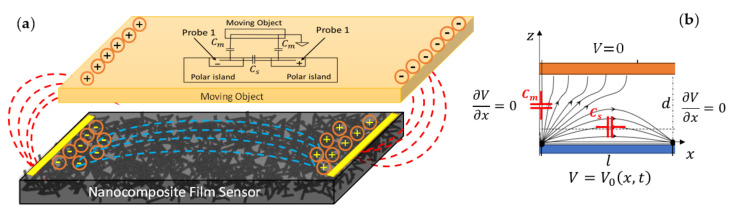
Schematic illustration of sensor mechanism for fringe-based TPU-CNT proximity sensor: (**a**) 3D model, charge distribution and fringe fields while object is in vicinity of the film sensor. (**b**) Symmetric cross section of the rectangular-shaped sensor model with cartesian coordinate system demonstrates boundary conditions [41].

To recognize the physics of the probing sensation, this sensing film (TPU-CNTs) constitutes two types of capacitors including a capacitor between neighboring probes (self-capacitance-C_s_) and fringe capacitance (mutual capacitance-C_m_), caused by the overlapping fringing field [40]. As shown in Figure 2a, when an object is quite far with respect to the film, the fringe capacitance between each probe with object is small. Approaching the probes, the fringe field between film and the sensing object turn out to be significant, (Figure 2b). In theory, shunting of the electric field changes the overall capacitance of the film. Hence, strength of electric field arising from the film capacitor is shifted and weakened by the object, thus reducing the stored charges in the film capacitor. Moreover, the mutual capacitance of the sensor is enhanced due to the reduced distance between probes and object while the capacitance of the actual film (self-capacitance) decreases owing to the change in the charge balance. Finding out an optimal nanocomposite sensor, different CNT contents were investigated earlier [40]. Absolute percentage change of capacitance to the initial capacitance at the different distance to the object was calculated and addressed as sensitivity. Moheimani et al. [41] demonstrated earlier that resistivity of the film sensor originating from CNTs amount can affect its sensitivity.

### 2.2. Experimental Work

#### 2.2.1. Material Preparation

TPU and MWCNT were used as the constituents of the nanocomposite sensors. A set of prototypes of film sensors were fabricated for different dilution of CNTs. To obtain TPU-CNT films with 0.5–0.6 mm thickness, TPU/CNT 10 wt% masterbatch nanocomposites, TPU/CNT 1–5 wt% pellets and filaments (twin-screw extruder) as well as a TPU/CNT hot press were prepared. Samples were cut out from the films in 60 × 20 mm squares. Figure 1 shows the major steps of the proposed method to process and manufacture the final product. Needless to say, the preparation of sensor samples can be carried out employing the 3D printing method.

#### 2.2.2. Sensor Read-Out and Characterization

While distances are ranging from 20 to 220 mm, the impedance analysis was performed by utilizing a probing station (Keithley 4200-SCS, Tektronix, Beaverton, OR, USA). Moreover, 60 × 20 mm nanocomposite coupons were compression molded (Carver 3946, Carver Inc., Wabash, IN, USA) into films of 0.5–0.6 mm in thickness from pellets. A brass bar (sensing object 10 mm height × 20 mm width × 200 mm length) approached the TPU/CNT sensor with a speed of 6.6 mm/s. To detect the maximum change in capacitance, the samples were pre-soaked with 5 V direct current to saturate and reduce the tunneling effect. Additionally, a 30 mV alternate current swiping signal was applied to measure the capacitance of the film with variable frequencies to achieve maximum stability of the working window for the fabricated sensors considering the energy consumption. Figure 1a,b represents the experimental setup and how change of capacitance to the initial capacitance (ΔC⁄C_0_) was analyzed. The full three sets of experiments were completed for each CNT content. Other conditions, including temperature and humidity, were strictly controlled to obtain an accurate measurement. The thin strips of both ends of the samples were then coated with conductive silver adhesive to erase the influence of contact resistance. The model parameters used for the tests are listed in Table 1.

## 3. Methodology

### 3.1. Governing Equations and Numerical Simulation

Schematic view of our proposed sensor along with a moving object and the fringe effect concept were shown in Figure 2. As observed, two-line electrodes are positioned at both sides of the nanocomposite substrate. When a voltage is applied between the electrodes, some current flows inside the substrate, resulting in charge distribution on the substrate. Furthermore, some fringing fields are developed outside the substrate which contribute to the stored charge on the nanocomposite surface. The current passing on the electrodes is monitored, and by dividing the current by the exciting voltage, the effective capacitance can be measured. While the object gets closer to the nanocomposite sensor, it distorts the fringing fields and consequently changes equivalence (self-capacitance). This characteristic of the system may be used to recognize the external object which is approaching the sensor. As Gauss’s law indicates, the amount of the stored charge on the right-hand side electrode decreases, and therefore, the measured capacitance declines too. Experimental measurements show this behavior of the system clearly [40]. In addition to experimental observations, an analysis was accomplished with a couple of goals by the authors [41]. Firstly, an analytical solution (simulation) helps to achieve a higher level of understanding regarding what happens in the system, and secondly, quantization of the changes of capacitance with respect to the distance of the object not only helps in calibration of the sensor but also is beneficial for the design of different sensors for various applications. Finally, this closed-form model (analytical simulation) is employed to optimize the sensing system.

Since there is no free stored charge in the part of space enveloped between the polymer substrate and the surface of object, the following equation governs the distribution of voltage in this region:

(1) $$\nabla^{2}V\left(x,y,z,t\right)=0$$

This solution was obtained by application of Gauss, conservation of charge, and Ohm laws on the Laplace equation. The total impedance seen by the LCR meter was calculated through dividing the applied voltage ($V_{exc}$) by the input current (i). The real part of derived impedance signified the equivalent resistance owing to the finite resistivity of the nanocomposite substrate while its imaginary part characterized the equivalent capacitance between the electrodes and object. Therefore, the following expression was used for calculation of the equivalent capacitance of the system [41]:

(2) Ceq=−ImiωVexc ,Req=−VexcRei

**Figure 3:**
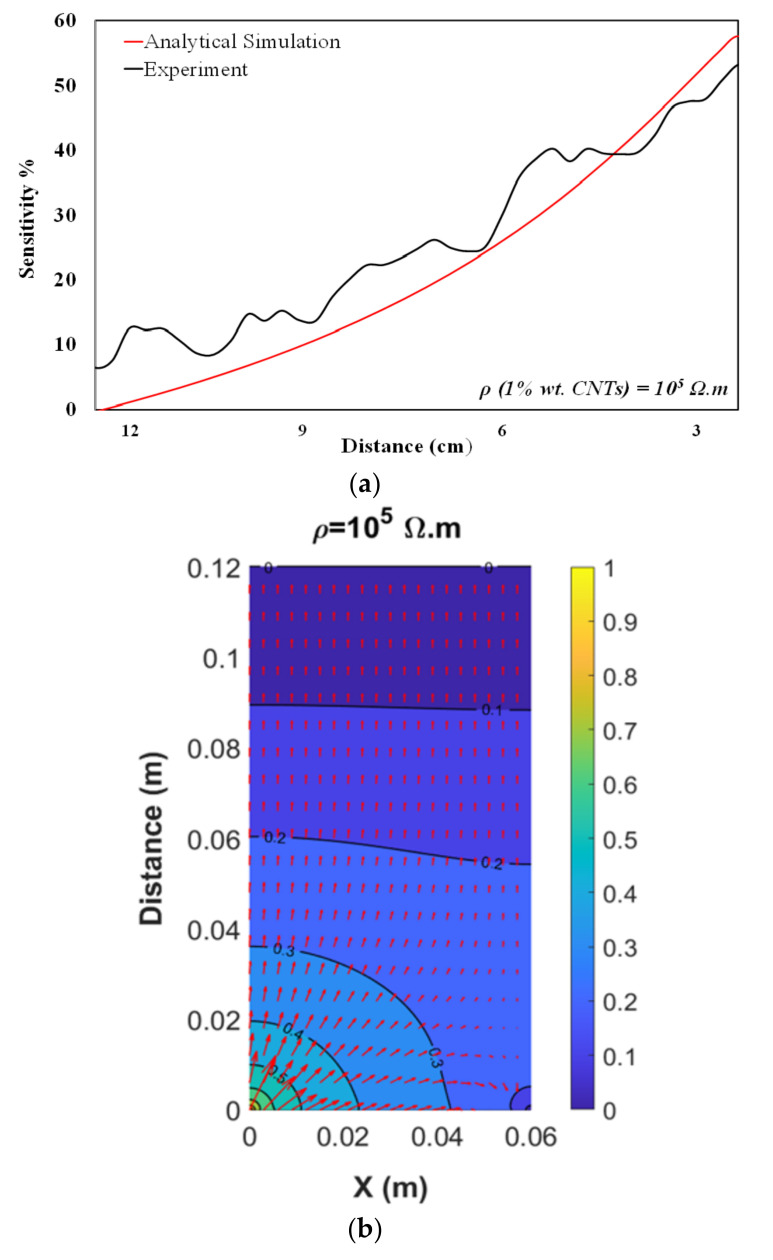
Sensing performance of the film sensor with 1% CNTs, ρ = 10^5^ Ωm. (**a**) Sensitivity (change of capacitance to the initial) comparison simulation result versus experimental one. (**b**) Potential contour for the in-phase voltages between probes and object at the distance of 12 cm.

A comparison of analytical and experimental models is plotted in Figure 3a over a distance of 9 cm (capacitance variation was normalized with respect to the initial capacitance). Both plots have displayed quite similar sensitivity behavior and reached to the peak of 0.5–0.6 at the distance of 3 cm. Apparently, the analytical model results are a bit larger than the measured ones. This discrepancy is primarily originated by the object used in the measurement. In the analytical model, the object dimension ought to be assumed infinite, contrary to that of the in situ object. Needless to say that the experiment is carried out for the distances shorter than 2–3 mm, but since the noise increases drastically and accuracy gets affected, we preferred to present the data with the highest signal to noise ratio. Moreover, our approach has sought a proximity sensor rather than a tactile sensor. Figure 3b illustrates the analytically calculated voltage distribution within the neighboring area between the sensor and object at certain values of resistivity (CNTs content). The graph’s horizontal axis shows the size of sensor length, and the vertical axis indicates the distance with the moving object. Furthermore, contour lines associated to certain values of the voltage are plotted together with vectors (in red) suggesting the magnitude and direction of the electric field at different points.

### 3.2. Artificial Neural Network (ANN)

ANN is among the best methods employed in machine learning (ML). It is created according to biological nervous systems and works based on the training–testing method. ANN is comprised of several components, namely, input, hidden, and output layers; neurons; and connections. In ANN, the network gathers data from previously solved problems to establish an arrangement of neurons that comprehend solving a new investigated problem. This method is extensively used to predict complicated functions with several inputs and outputs which causes a considerable reduction in solving time.

The optimization process seeks the feasible domain of variables and then finds the objective functions in each iteration so as to find the optimum design variables. Considering multiple design variables and two objective functions as well as employing the closed-form simulation (analytical model) make the process time-consuming. Using an accurate and fast approximation method increases the efficiency of the process and considerably reduces the optimization time. In the current work, an Artificial Neural Network (ANN) is employed as a tool to achieve sensitivity of sensor based on previously obtained results.

**Figure 4:**
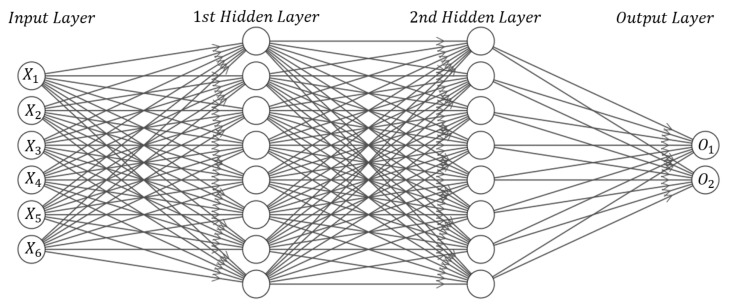
Schematic of implemented Neural Network for black-box modeling (with 1000 samples).

In this study, a Multi-Layers Perceptron (MLP) network with layers, two hidden and one output layers, is defined. The training is accomplished by the Levenberg–Marquardt backpropagation training function. In the training procedure, minimization of Root Mean Square Error (RMSE) between the outputs and targets of the training set is considered as the criterion to evaluate the network performance. Figure 4 describes the design of neural network. x_1_ to x_6_ represent the defined design variables, and O_1_ to O_2_ are the network’s outputs, namely, sensitivity and cost. Table 2 shows the domain (the lower and upper limits) of all design variables. For (L) and (b), the limits were chosen so that they could be easily cut from the fabricated film sheets while still maintaining the assumption that the length to width ratio does not go beyond five. Excessive upper bounds that would lead to very big devices were also avoided. For (R), the lower bound is to ensure there is sufficient reinforced CNTs to make the dielectric semi-conductive while the upper bound is to avoid an excessive amount of current which is not feasible and consumes energy. For (h), the lower bound is the least possible thickness of a easily available dielectric sheet while the upper bound ensures that the thickness can be altered to match the other parameters as much as possible because it is unlikely to ever go beyond this value for the chosen resistance value. A 1000-samples data set is provided by the MATLAB analytical script. The inputs of these samples are generated randomly in the explained range in Table 2. This data set is entered into the ANN as the training data in order to comprehend the relationship between the inputs and the output. The architecture of the utilized ANN including the number of neurons and transfer functions of each layer is selected based on the latest studies [42,43] and is described in Table 3.

### 3.3. Genetic Algorithm Optimization

The purpose of optimization process is to find the optimum range or value for objective functions and also to determine the corresponding value of decision variable in which the optimum points are attained. Since the fitness function of this study is highly non-linear, the heuristic optimization methods have priority in the optimization process [33]. The Genetic Algorithm as one of the evolutionary optimization methods is one of the most popular methods for optimizing complicated models. In this research, the non-dominated Genetic Algorithm (NSGA2) toolbox of MATLAB is used to optimize the proposed model. As mentioned in the previous section, the fitness function of optimization is the output network of ANN which has an acceptable runtime. The runtime of the analytical model is around 10 min; however, the runtime of the ANN black-box model is around 13 ms.

**Figure 5:**
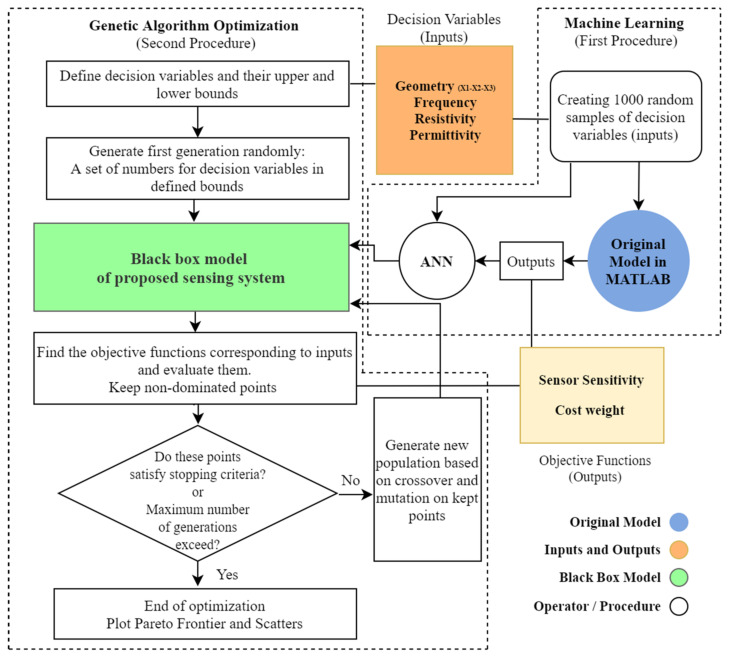
The flowchart of ANN and Genetic algorithm process and connections.

Figure 5 exhibits the flowchart of optimization and machine learning procedures. Accordingly, first, the machine learning process is accomplished by training a black-box model using one thousand samples as inputs and outputs, and then, the black-box model is considered the fitness function of the genetic algorithm to find the optimum solutions for sensitivity and cost of the proposed system.

## 4. Results and Discussion

### 4.1. ANN Results

**Figure 6:**
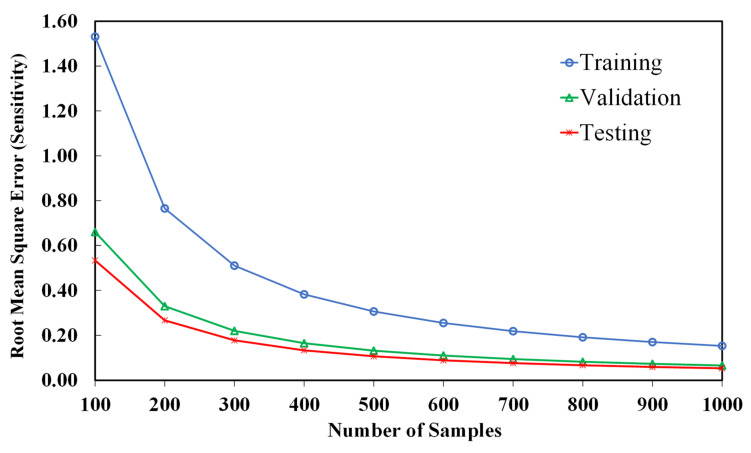
The effect of the number of samples on the RMSE of training, validation, and test sets.

The Artificial Neural Network (ANN) has shown a perfect match with the original model. The root mean square errors (RMSE) of sensitivity for a different number of samples are calculated in order to show that the 1000 samples are enough for training an accurate network. Figure 6 illustrates the training, validation, and testing errors of sensitivity for different numbers of samples from 100 to 1000. This figure, which is also known as the error elbow figure, indicates that a higher number of samples than 600 is sufficient for training a neural network for the present data set. It should be noted that Figure 6 shows the ANN results for CNT of 1.25% wt., and in this study all the 1000 samples are used to attain the highest accuracy. Earlier in Table 3, the characteristics of the ANN were presented. Accordingly, a network with two hidden layers containing 8 neurons shows a sufficient accuracy. The maximum relative network errors observed in the training were 0.56% and 2.89% for sensitivity and cost, respectively.

### 4.2. Optimization Results for 1.25% CNT

**Figure 7:**
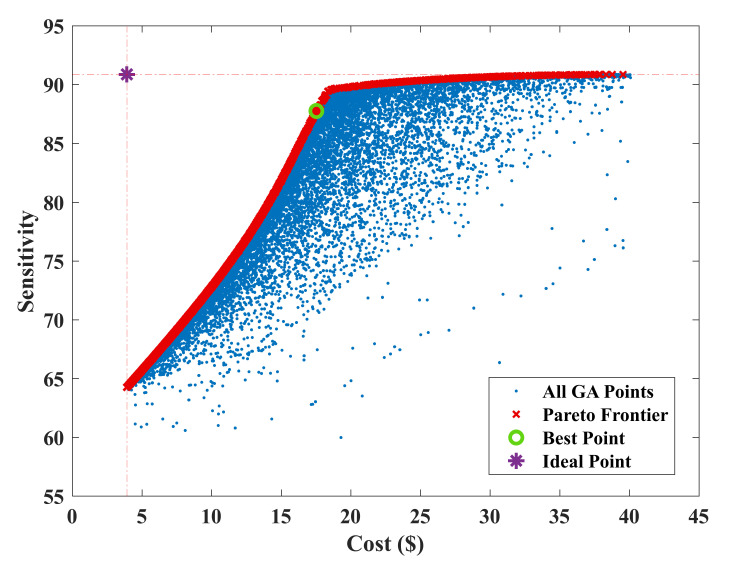
Pareto Frontier of cost and sensitivity as a result of optimization for nanocomposite sensor with 1.25% wt.

**Figure 8:**
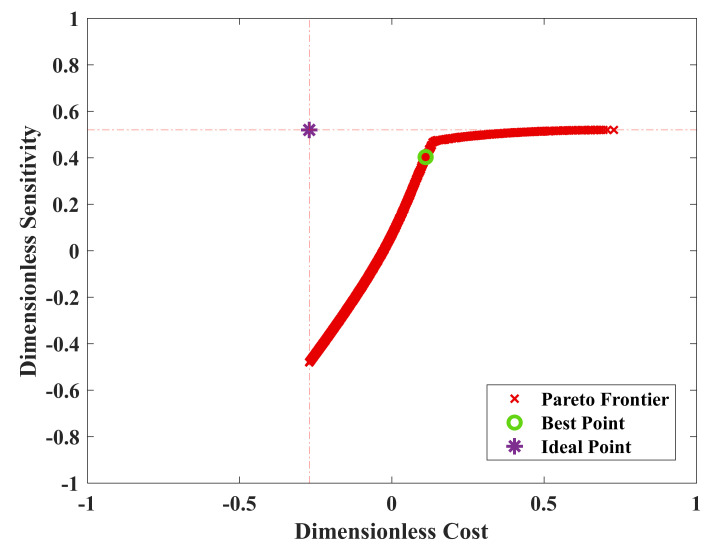
Dimensionless Pareto Frontier of cost and sensitivity as a result of optimization for nanocomposite sensor with 1.25% wt.

The result of sensitivity and cost dual-objective optimization of film sensors were reinforced with 1.25% wt. CNT is presented in Figure 7. As it can be seen, every point has a special input vector and two objective values, cost and sensitivity. The blue points show all the 1000 generated points by the Genetic Algorithm which are called dominated points. The red points are the non-dominated optimum solutions which are also known as Pareto Frontier. The purple point is the ideal point which has the maximum sensitivity and minimum cost. The ideal point is not a feasible solution; however, it can be used for finding the best point of the Pareto Frontier. The best point of the Pareto Frontier is the closest point to the ideal point in a dimensionless environment which is presented in Figure 8. The Genetic Algorithm searches the area inside the bounds which are presented in Table 2 to find the prominent members of the input vectors with the maximum sensitivity at the minimum given cost. Among all solutions, the Pareto-frontier curve illustrates the optimal solutions for the optimization problem. Each point of this curve defines a design variables vector, (x_1_…x_6_), which in turn has the maximum sensitivity for a corresponding cost. According to Figure 7, the ideal point has a sensitivity of 91% and a cost of USD 4.7. The best point has a sensitivity of 87.02% and a cost rate of USD 18.14.

### 4.3. Optimization Results for all CNTs

**Figure 9:**
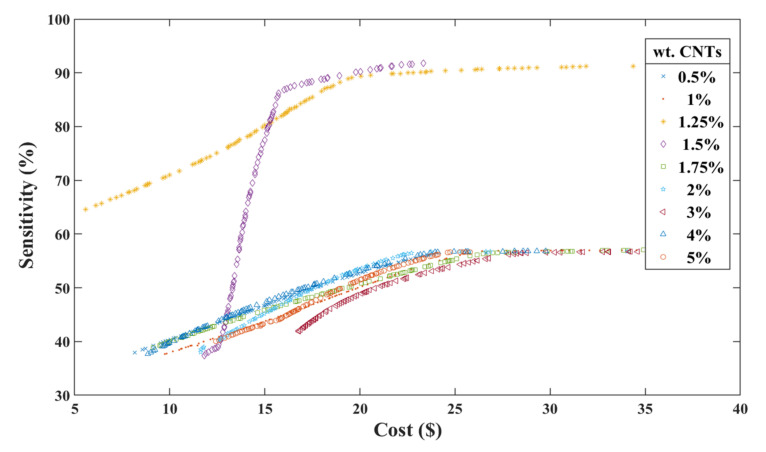
2D Pareto Frontier of sensitivity and cost of active materials, for different CNTs %wt.

Regarding the CNTs’ functionality as the building blocks of the sensor, the behavior of other film proximity sensors with different CNTs weight through a distance of 24 cm has been depicted in Figure 9. Earlier, it was shown that changing the percent of active materials (CNTs) used in the film sensor can significantly affect the sensitivity of the sensor [40]. Therefore, a comprehensive study on the other CNTs percentage (resistivity) was needed.

As expected, Figure 9 demonstrates that the highest sensitivity occurs between 1–2% CNTs, and it entirely follows our previous studies [40,41]. They showed that the highest sensitivity at the certain distance occurs around the percolation threshold where the conductive pathways are fully constructed. A Pareto Frontier of 1.25% and 1.5% CNTs exhibits a big shift from others. However, a film sensor with 1.5% CNTs illustrates a sharp slop at the beginning, and the cost range is more limited in respect to other plots.

With this in mind, the detailed values for decision variables and objective functions of best points are specified and depicted in Table 4. On the right side of the table, cost, ANN, and analytical (simulation) sensitivity are listed. As Table 4 shows, approximate (ANN) results are in acceptable agreement with the analytical results. Geometry-related design variables (X_1_, X_2_, X_3_) of the best points for different CNTs percentage are almost in the same range. However, a wider range of change can be seen for property-related design variables (X_4_, X_5_, X_6_). Hence, there is an agreeable certainty on the optimized geometry for all the film sensors. As a result of that, thickness, length, and width of about 0.5, 90, and 20 mm can be selected, respectively. On the other hand, the value of property-based design variables such as frequency and system impedance does not have a significant pattern. For instance, in a film sensor with 1.25% and 1.5% CNTs, frequency and impedance show quite low magnitudes compared with other weights of CNTs. It should be mentioned that a lower value of frequencies also results in energy usage reduction (about one-hundredth of other CNTs weight).

### 4.4. Scatters of Distribution for 1.25% CNT

**Figure 10:**
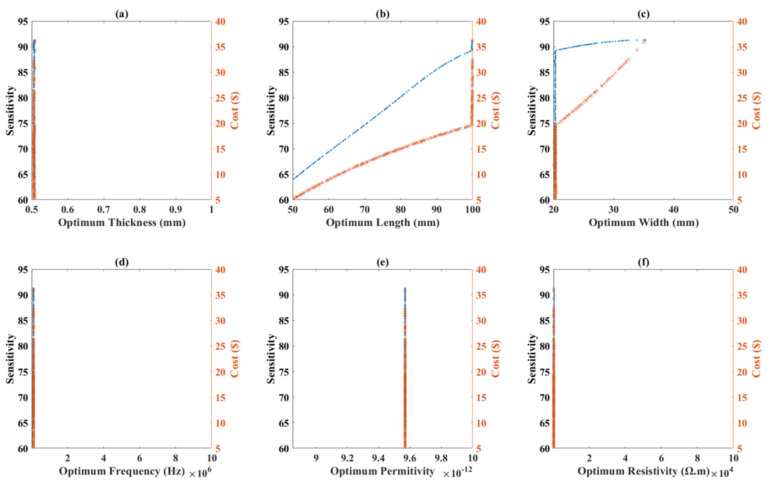
(**a**–**f**) Scatter of distribution for different design parameters versus objective functions as a result of optimization for a sensor with 1.25% wt. CNTs.

Figure 10 illustrates the scatters of distribution for the different optimum design parameters with corresponding value for the objective functions. In this figure, the optimum value of design parameters for Pareto Frontier are presented in the order of the population in which they are found. Using this figure, researchers can obtain the best range for decision variables in order to have a robust sensor in terms of sensitivity and cost. Evaluating Figure 10a,d demonstrates that all cases nearly take the minimum feasible value in the domain. However, regarding Figure 10b, length captures mostly the maximum possible value in the domain, and the sensor performance is thought to be most affected by the length. The longer the sensor is made, the better the sensitivity achieved. Therefore, this is mainly to increase the volume and subsequently the cost which also increases with the smaller slop and finally reaches a maximum cost. Hence, an optimized proximity sensor can be achieved with the fabrication of a thin strip film with a narrow width. This scatter plot also gives a solid perception on the properties effect. Figure 10d shows that the operating frequency does not need to exceed 100 kHz to reach the optimum point. The smaller the frequency the sensing system needs, the lower the amount of energy it consumes. Regarding the permittivity and resistivity, the optimum point is obtained in the mid-range.

### 4.5. Parametric Studies of Different Parameters on Sensitivity

**Figure 11:**
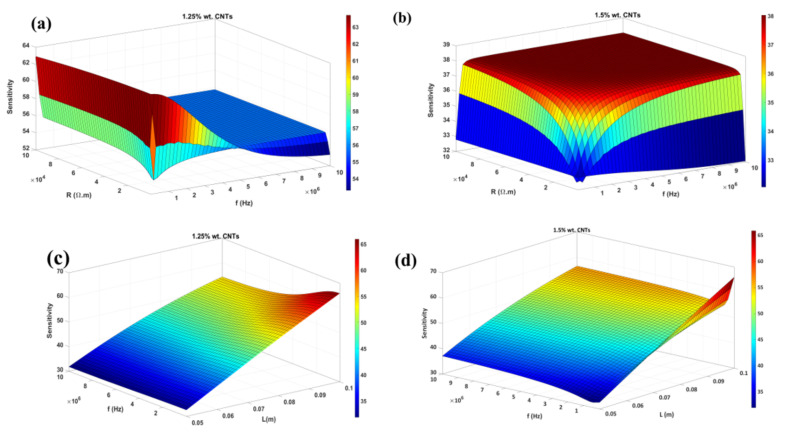
3D effect of different input parameters on shape of objective function (sensitivity): (**a**) frequency and resistivity for 1.25% wt. CNTs (**b**) frequency and resistivity for 1.5% wt. CNTs (**c**) frequency and length for 1.25% wt. CNTs (**d**) frequency and length for 1.25% wt. CNTs.

To illustrate the complexity of accurately assessing the direct impact of two input parameters at their bound, a 3D surface can be utilized to denote the objective function while the other parameters are varied. The sensitivity results and the most important parameters influencing the performance of the sensing device are illustrated in Figure 11. This section mostly focuses on the two best Pareto Frontiers which noticeably show a higher range of sensitivity. It is obvious that the objective function does not form a clearly convex shape for a film sensor with 1.25% wt. CNTs; however, there is a complete dome for one with 1.5% wt. CNTs (Figure 11b). Furthermore, higher applied frequencies do not provide necessarily a better sensitivity. For both CNTs percentages, the maximum point could occur at the lower frequencies and similar resistivities. Needless to say, higher frequency and resistivity demand an infinite amount of current, which is very costly. Having lower amounts of these parameters gives the manufacturer the certainty that energy consumption is seriously addressed in the fabrication process. In addition, the effect of frequency and length can be observed simultaneously in the sensitivity for both CNTs weight in the photo. The sensitivity grows quite linearly with these two changes over their domain. As expected, frequency becomes noticeable in those longer film sensors. Lastly, we manufactured 1.25% wt. CNTs and did the experiment for the optimum value to have further confirmation with the data obtained from the simulation. The cut-out film sensor showed a sensitivity of 85.92% with around 1% difference with ML.

## 5. Conclusions

In the current research, the multi-objective optimization of a nanocomposite proximity sensor was conducted considering two objective functions, namely, sensitivity and cost. Six design variables including thickness, width, length, frequency, permittivity, and impedance of the film sensor were taken into account. The sensory system was manufactured, tested, and later analytically modeled in MATLAB. Running the analytical model is a time-consuming procedure since every run of Fourier series should be solved for at least 100 nodes, which takes 20 min. The optimization process requires thousands of runs, and using the original model was impossible. An artificial neural network was used to simulate the sensing process and reduce the solution time. Utilizing the GA method, the maximum sensitivity load for each given CNTs percentage was obtained, and the Pareto-frontier curve was generated. Results demonstrate that the thickness of the film sensor and its width tend to reach determined low values during the optimization process. On the contrary, the sensor length follows a linear trend, taking maximum values among optimum solutions. As expected, the nanocomposite sensor reaches the maximum sensitivity at the specific range of nanoparticle contents (1.0–1.5% CNTs). Moreover, the results show that almost all of the optimum points on the Pareto-frontier curve have minimum thickness and frequency, which illustrates the importance of the thickness related to the other design variables.

## Figures and Tables

**Table 1:** Experiment testing parameters and setup.

Property (Symbol)	Value
TPU Density	1.12 (g/cm^3^)
MWCNT Density	1.75 (g/cm^3^)
Cost MWCNTs	20 $/grm
Dielectric Thickness	0.5 (mm)
Vacuum Permittivity	8.8542 (pF/m)
Film Sensor Resistivity	1–10^5^ (Ωm)
Frequency	100–1000 (kHz)
AC Voltage	30 mV
DC Voltage	5 V
Object’s speed	6.6 mm/s

**Table 2:** Domains and characteristics of the variables.

Variable	Design Parameters	(Symbol)	Lower Bound Min.	Upper Bound Max.
X_1_	Device Thickness	*h* (mm)	0.5	1
X_2_	Device Length	*L* (mm)	50	100
X_3_	Device Width	*b* (mm)	20	50
X_4_	Frequency	*f* (Hz)	10^3^	10^7^
X_5_	Dielectric Relative Permittivity	*ε_r_*	1	8
X_6_	Impedance (resistivity)	*Ω* (Ohm·m)	10^2^	10^5^

**Table 3:** The architecture of ANN.

1st Hidden Layer		2nd Hidden Layer		Output Layer		Training Samples	Testing Samples	Validating Samples
neurons	Transfer fn.	neurons	Transfer fn.	neurons	Transfer fn.	80%	10%	10%
8	Tangent sigmoid	8	Tangent sigmoid	2	Linear			

**Table 4:** Detailed value for decision variables and objective functions of Pareto Frontier best points for different CNTs %wt.

		Inputs						Outputs			
GA Runtime (s)	CNTs %wt.	X_1_ (10^−4^)	X_2_	X_3_	X_4_ (10^6^)	X_5_ (10^−12^)	X_6_ (10^4^)	Predicted by AAN		Calculated by Simulation	
								Sensitivity %	Cost USD	Sensitivity %	Cost USD
221	0.5	5.012	0.0926	0.0201	1.2953	9.991	1.4463	54.05	21.014	54.53	20.94
294	1	5.0955	0.0857	0.0201	8.82	9.8964	6.0655	52.65	22.026	52.64	22.75
241	1.25	5.0658	0.0935	0.0202	0.096978	9.567	0.03092	87.02	18.14	84.70	21.49
326	1.5	5.2305	0.0503	0.0201	0.091565	9.2711	0.2268	85.35	15.64	83.12	16.89
271	1.75	5.0181	0.09199	0.02005	1.34906	9.99127	1.73746	54.00	20.96	54.39	20.85
327	2	5.0218	0.0944	0.02101	5.8707	9.1241	5.8509	55.24	21.37	55.18	21.51
324	3	5.2225	0.0956	0.0212	4.8568	9.1334	6.3342	56.35	23.06	56.45	22.71
282	4	5.2519	0.0977	0.0200	6.9567	9.8529	4.1619	56.07	23.07	56.07	23.38
256	5	5.1270	0.0782	0.02004	4.04022	9.7919	5.3694	50.12	19.108	50.14	18.34

## Data Availability

Not applicable.
